# Association between duration of progesterone supplementation and clinical outcomes in artificial frozen-thawed embryo transfer cycles

**DOI:** 10.3389/fendo.2023.1193826

**Published:** 2023-07-27

**Authors:** Ling Liu, Hongyan Zhou, Jie Hu, Xingyu Sun, Doudou Liu, Guiying Huang

**Affiliations:** ^1^ Reproductive Medicine Center, The Affiliated Hospital of Southwest Medical University, Luzhou, China; ^2^ Department of Gynecology, The Affiliated Traditional Chinese Medicine Hospital of Southwest Medical University, Luzhou, Sichuan, China

**Keywords:** artificial endometrial preparation, endometrial transformation time, frozen-thawed embryo transfer (FET), implantation rate, pregnancy outcome, window of implantation (WOI)

## Abstract

**Objective:**

The administration of progesterone before transfer in hormone replacement treatment (HRT) is crucial for the clinical outcomes of frozen-thawed embryo transfer (FET), but the optimal duration of progesterone remains controversial. This study aimed to investigate the effect of the duration of progesterone administration on the clinical outcomes of FET cycles.

**Methods:**

This prospective cohort study included 353 artificial FET cycles conducted at a reproductive medicine center between April and October 2021. The FET cycles were stratified into four groups based on the duration of progesterone supplementation before the procedure and the embryonic development stage: group P3 (73 patients) received intramuscular progesterone for 3 days and group P4 (87 patients) for 4 days before Day 3 frozen embryo transfer, group P5 (70 patients) for 5 days and group P6 (123 patients) for 6 days before frozen blastocyst transfer. This trial was performed using one or two vitrified embryo(s) when the endometrial thickness reached 7 mm after estrogen supplementation in an artificial cycle. The primary outcome was clinical pregnancy, and secondary outcomes included biochemical pregnancy, implantation, early pregnancy loss, and live births.

**Results:**

There were no significant differences in the demographic and clinical characteristics between the groups. No significant difference was observed in the clinical pregnancy rates between groups: 23/73 (31.5%) in group P3 vs 28/87 (32.2%) in group P4 (*P* = 0.927). Compared to group P5 (41/70, 58.6%), the clinical pregnancy rate was not significantly different in group P6 (77/123, 62.6%, *P* = 0.753). There was no significant difference in the implantation rates between groups: 33/136 (24.3%) in group P3 vs 34/166 (20.5%) in group P4 (*P* = 0.431), and 62/133 (46.6%) in group P5 vs 107/231 (46.3%) in group P6 (*P* = 0.956). The duration of progesterone supplementation (mean: 3.5 ± 0.5 days; range:3–4 days) before Day 3 frozen embryo transfer did not impact clinical pregnancy (odds ratio [OR] 1.048; 95% confidence interval [CI], 0.518–2.119). The duration of progesterone administration (mean: 5.6 ± 0.5 days; range:5–6 days) before frozen blastocyst transfer may not affect clinical pregnancy (OR 1.339; 95% CI, 0.717–2.497).

**Conclusion:**

There may be no significant correlation between the duration of progesterone supplementation and pregnancy outcomes in artificial FET cycles, although the clinical pregnancy rate was higher when progesterone supplementation was extended for one day before FET.

## 1 Introduction

Due to recent developments in clinical practice and laboratory technology, embryo cryopreservation has become a central component of assisted reproductive technology (ART). Improved laboratory technology has contributed to an increased number of embryos available for *in vitro* fertilization (IVF) or intracytoplasmic sperm injection (ICSI) cycles. The vitrification method, improved embryo survival rates after thawing (1, 2), and implementation of a single embryo transfer policy to reduce multiple pregnancies without reducing the cumulative delivery rate (3), have boosted the number of FET cycles performed worldwide. A freeze-all strategy is now adopted for a growing number of indications, including the prevention of ovarian hyperstimulation syndrome, preimplantation genetic testing (PGT), progesterone elevation in the late follicular phase, endometrial abnormality, embryo–endometrium asynchrony, egg freezing, and fertility preservation, and so on (4–11). As a result, the proportion of autologous frozen embryo transfers has increased dramatically worldwide over the last decade, which is of great significance in improving the clinical outcomes of FET (12).

Despite the emerging importance of the FET cycle, optimal endometrial preparation before it remains unclear. Although several randomized trials have examined the effects of different cycle regimens on FET, there is still no evidence for a single optimal endometrial priming protocol for FET cycles (13–16). Hormone replacement therapy (HRT) is currently the most widely administered therapy due to its wide range of applications, low cycle cancellation rate, and no requirement for frequent follow-ups (17). Patients can participate in the HRT program irrespective of their ovarian functional status and menstrual irregularities (18). Several studies have suggested that HRT cycles are comparable to natural cycles in terms of pregnancy, miscarriage, and live birth rates (14–16). Most reproductive medicine centers primarily use HRT in endometrial preparation for various reasons. In artificial FET cycles, estrogen and progesterone are sequentially implemented to synchronize the embryo transfer with the window of implantation (WOI) (19). Supplementation of estrogen before FET promotes endometrial thickening, and estrogen is continued as a daily supplementation of progesterone, which is initiated a few days before scheduled embryo transfer. There are two significant factors for successful implantation and pregnancy, namely euploid embryos with developmental potential and a synchronous endometrium (20).

In contrast to estrogen, progesterone appears to be a significant determinant of the WOI because the endometrial WOI is confined to the stenosis interval in the luteal phase (19, 21). Endometrial WOI was first characterized by studies applying hormone preparations in recipients with donor oocytes, indicating that endometrial receptivity is significantly reduced when the frozen embryo is administered prior to or after this critical period (22, 23). Therefore, determining the optimal duration of progesterone exposure before FET is crucial for maximizing the success of ART (24). No clear and definite conclusion regarding the superiority of one protocol over another can be drawn thus far. Most researchers maintain that a stable clinical pregnancy rate can be obtained by transferring the 3rd day (Day 3) embryos with progesterone supplementation for 3 days and blastocysts with progesterone administration for 5 days in the FET cycle (25, 26). However, there are always some unavoidable factors that lead to the incomplete synchronous development of the embryos and endometrium (27, 28). Although some previous studies have indicated that appropriately delaying the transfer time may improve the outcomes of FET (29), another data did not show any difference in clinical pregnancy rates between protocols with or without prolonged progesterone supplementation before FET (30).

As for the lack of evidence regarding to the optimal duration of progesterone administration, there is still much more to be learned about endometrial preparation and synchronization to maximize endometrial function and receptivity, and select the best time for embryo transfer (31). This study assessed whether the duration of progesterone administration before FET influenced the clinical outcomes of HRT of during the FET cycle.

## 2 Materials and methods

### 2.1 Study design and participants

This prospective study was conducted at the Reproductive Medical Center of the Affiliated Hospital of Southwest Medical University. This study included patients who underwent FET in an artificial cycle from April 2021 to October 2021 and were allocated to one of four groups as soon as the endometrial thickness reached 7 mm after estrogen supplementation. One or two embryo(s) were transferred according to Health Commission legislation. The inclusion criteria were patients aged 22−45 years and on hormone replacement cycles. The exclusion criteria included known allergic reactions to progesterone products, uptake of an experimental medicine within 30 days before study initiation, and cancellation of FET for various reasons. The cycles were stratified into four groups based on the duration of progesterone supplementation before embryo transfer and the embryonic development stage. The cycles were assigned to groups P3 or group P5 from April 2021 to June 2021. The cycles were assigned to Groups P4 and P6 from July 2021 to October 2021. Patients in groups P3 and P4 were administered intramuscular progesterone for 3 and 4 days, respectively, before frozen-thawed Day 3 cleavage-stage embryo transfer. Patients in groups P5 and P6 were separately supplemented with intramuscular progesterone for 5 and 6 days, respectively, before the frozen-thawed blastocyst transfer. This trial was performed using one or two vitrified-warmed embryo(s) when the endometrial thickness reached 7 mm after estrogen supplementation in an artificial cycle. This study was approved by the Institutional Review Board of the hospital. Written informed consent was obtained from all participants.

### 2.2 Endometrial preparation and embryo transfer

All cycles were performed using the same artificial protocol. Oral estradiol valerate (Progynova, BayerSchering Pharma AG, Germany) was administered on the second or third day of the menstrual cycle after verifying that the patients were in the early proliferative phase of the menstrual cycle. Typically, 3 mg of estradiol valerate was prescribed twice daily. If the endometrial thickness was <7 mm 14 days later, patients were required to comply with a step-up protocol that involved the addition of vaginal estrogen supplementation (Femoston, Abbott Healthcare Products; 1 mg once daily), to the oral administration of estradiol valerate, based on the physician’s preference and experience. Serum estradiol and progesterone levels were determined on the day before the initiation of progesterone treatment.

Intramuscular progesterone (60 mg once a day) was initiated, supplemented with oral dydrogesterone (10 mg thrice a day) when the endometrial thickness was 7 mm. Embryo transfer was performed days 4, 5, 6 and 7 of progesterone exposure in groups P3, P4, P5 and P6 respectively. No more than two embryos were transferred in any FET cycles. All the embryos were thawed on the morning of the transfer. Embryos were evaluated according to the conventional classification system currently used in our IVF laboratory: number of blastomeres, degree of cytoplasmic fragmentation and the equality of blastomeres (32). Embryos with seven to nine cells on Day 3 and with no multinucleation, cytoplasmic fragmentation, or less than 10% fragmentation were considered good-quality embryos. The blastocyst morphology was evaluated according to the Gardner and Schoolcraft grading system (33). A morphological quality score was assigned to the blastocysts at the moment of transfer; blastocysts with inner cell mass/trophectoderm (ICM/TE) score types AA, AB, or BA were considered good-quality embryos (30). The daily estrogen and progesterone protocol was continued if there was a negative pregnancy test result after FET. If pregnancy was achieved, hormone administration was continued until approximately 11–12 weeks of gestation.

### 2.3 Outcome measures

The independent variable of interest was the duration of progesterone exposure, defined as the number of days from the initiation of progesterone treatment to the completion of embryo transfer. To evaluate whether the duration of progesterone administration before FET affected the clinical outcomes, the primary outcome of the study was clinical pregnancy, which was defined as the presence of one or more gestational sac(s) with an embryonic pole indicating the fetal heartbeat on transvaginal ultrasound at 7 weeks of gestation. Secondary outcomes included biochemical pregnancy, rate of implantation, live births, early pregnancy loss, and miscarriage.

Fourteen days after embryo transfer, having a serumβ-hCG level of >5 IU/L was considered a biochemical pregnancy. The implantation rate was determined by dividing the number of intrauterine sacs inspected using transvaginal ultrasound divided by the number of embryos transferred. Live birth was defined as the delivery of viable infant(s) at ≥24 gestational weeks. Early pregnancy loss was considered when no gestational sac was observed even after a serum β-hCG ≥ 5 mIU/mL or loss occurring after the presence of an intrauterine gestational sac was confirmed.

### 2.4 Statistical analysis

Normally distributed measurement data are presented as the “mean ± standard”; data that is not normally distributed are presented as “median (range).” The Shapiro- Wilk (SW) test was used to determine whether the continuous variables were normally distributed. Statistical comparisons between the two groups were performed using the Mann-Whitney U test. Categorical variables were described as frequencies or percentages, and comparisons between groups were performed using Pearson’s chi-squared test or Fisher’s exact test. All statistical analyses were performed using IBM SPSS Statistics for Windows (version 19.0; IBM, Corp., Armonk, NY, USA. Statistical significance was set at *P* < 0.05.

## 3 Results

Overall, 353 FET cycles were performed with HRT were analyzed in this study, including FET of cleavage embryos or blastocysts. All FET cycles were stratified into four groups (P3, P4, P5, and P6) according to the embryonic development stage and the duration of progesterone administration before FET. At the time of analysis, groups P3, P4, P5 and P6 were compared.

### 3.1 Baseline characteristics

Comparison of baseline demographic and cycle characteristics based on the duration of progesterone administration before frozen-thawed Day 3 cleavage-stage embryo transfer. The baseline characteristics are presented in **Table 1**. There were no significant differences in the baseline characteristics between groups P3 and P4. Comparison of baseline demographic and cycle characteristics according to the duration of progesterone exposure before frozen blastocyst transfer was performed. There were no significant differences in demographic and clinical characteristics between groups P5 and P6 (**Table 2**).

**Table 1:** Baseline and cycle characteristics for frozen-thawed day 3 embryo transfer cycles.

Parameters	Group P3 (n=73)	Group P4 (n=87)	*P*
Female age (year)	32.4 ± 5.8	33.3 ± 5.4	0.314^a^
Male age (year)	34.2 ± 6.3	35.2 ± 5.9	0.291^a^
Previous conception (%)	33(45.2%)	30(34.5%)	0.167^b^
Years of infertility	5.9 ± 4.0	5.5 ± 4.6	0.530^a^
BMI (kg/m2)	22.9 ± 3.5	23.2 ± 3.1	0.648^a^
Basic FSH(mIU/ml)	9.2 ± 4.5	10.0 ± 5.0	0.279^a^
Basic LH(mIU/ml)	4.1 ± 2.8	4.4 ± 2.8	0.502^a^
Basic E2(pg/ml)	35.3 ± 15.3	32.9 ± 16.9	0.340^a^
Basic PRL	13.4 ± 8.2	14.4 ± 8.9	0.447^a^
Basic P	0.7 ± 0.5	0.6 ± 0.5	0.445^a^
AMH(ng/ml)	3.3 ± 3.2	2.9 ± 3.3	0.352^a^
AFC	16.8 ± 9.3	16.3 ± 9.2	0.757^a^
Endometrial thickness (mm)	9.7 ± 1.9	9.8 ± 2.2	0.822^a^
No. of embryos transferred	1.9 ± 0.4	1.9 ± 0.3	0.425^a^
No. of good-quality embryos transferred	1.4 ± 0.7	1.4 ± 0.7	0.554^a^
E2 level on the day of planning FET	543.6 ± 515.4	490.3 ± 468.4	0.494^a^
P level on the day of planning FET	53.6 ± 12.1	52.7 ± 12.9	0.678^a^
Concomitant infertility factors			
Tubal factor (%)	49(67.1%)	59(67.8%)	0.926^b^
Ovulation disorder (%)	11(15.1%)	12(13.8%)	0.819^b^
Decreased ovarian reserve (%)	4(5.1%)	7(8.0%)	0.523^b^
Male factor (%)	9(12.3%)	9(10.3%)	0.692^b^
Length of estradiol supplement	17.8 ± 4.3	18.4 ± 4.7	0.436^a^

^a^Mann–Whitney U test, ^b^Pearson’s Chi squared test.

BMI, body mass index; FSH, Follicle stimulating hormone; LH, Luteinizing hormone; E2, estrogen; PRL, prolactin; P, progesterone; AMH, anti-Müllerian hormone; AFC, antral follicle count.

**Table 2:** Baseline and cycle characteristics for frozen-thawed blastocysts transfer cycles.

Parameters	Group P5 (n=70)	Group P6 (n=123)	*P*
Female age (year)	30.5 ± 4.1	31.4 ± 4.5	0.187^a^
Male age (year)	32.0 ± 4.3	33.4 ± 6.0	0.058^a^
Previous conception (%)	37(52.9%)	49(39.8%)	0.080^b^
Years of infertility	5.2 ± 3.8	5.1 ± 3.5	0.918^a^
BMI (kg/m^2^)	22.8 ± 3.2	22.6 ± 3.5	0.721^a^
Basic FSH(mIU/ml)	7.9 ± 1.7	8.3 ± 2.3	0.247^a^
Basic LH(mIU/ml)	4.7 ± 3.0	5.3 ± 5.1	0.431^a^
Basic E2(pg/ml)	43.0 ± 35.2	35.3 ± 27.3	0.090^a^
Basic PRL	12.9 ± 6.6	14.1 ± 7.8	0.286^a^
Basic P	0.6 ± 0.6	0.6 ± 0.4	0.634^a^
AMH(ng/ml)	5.1 ± 4.1	4.9 ± 4.1	0.661^a^
AFC	23.3 ± 11.1	22.6 ± 10.1	0.633^a^
Endometrial thickness (mm)	10.4 ± 2.4	10.1 ± 2.4	0.473^a^
No. of embryos transferred	1.9 ± 0.3	1.9 ± 0.3	0.647^a^
No. of good-quality embryos transferred	1.5 ± 0.8	1.3 ± 0.8	0.184^a^
E2 level on the day of planning FET	503.5 ± 476.3	556.0 ± 569.0	0.515^a^
P level on the day of planning FET	54.1 ± 12.3	55.9 ± 10.4	0.285^a^
Concomitant infertility factors			
Tubal factor (%)	40(57.1%)	87(70.7%)	0.056^b^
Ovulation disorder (%)	11(15.7%)	13(10.6%)	0.298^b^
Decreased ovarian reserve (%)	2(2.9%)	1(0.8%)	0.270^b^
Male factor (%)	17(24.3%)	12(9.8%)	0.014^b^
Length of estradiol supplement	18.0 ± 4.6	18.5 ± 4.3	0.487^a^
Day 5 blastocysts(%)	56(80.0%)	104(84.6%)	0.419^b^

^a^Mann–Whitney U test, ^b^Pearson’s Chi squared test.

BMI, body mass index; FSH, Follicle stimulating hormone; LH, Luteinizing hormone; E2, estrogen; PRL, prolactin; P, progesterone; AMH, anti-Müllerian hormone; AFC, antral follicle count.

### 3.2 Pregnancy outcomes

Pregnancy outcomes stratified into two groups according to the duration of progesterone administration prior to frozen-thawed Day 3 cleavage-stage embryo transfer are listed in **Table 3**. No significant differences were observed between groups in the rates of clinical pregnancy, biochemical pregnancy, implantation, live birth, early pregnancy loss, and miscarriage. The pregnancy outcomes stratified into two groups according to the duration of progesterone exposure before frozen blastocyst transfer are listed in **Table 4**. There were no significant differences between groups in the rates of clinical pregnancy, biochemical pregnancy, implantation, live birth, early pregnancy loss rate, or miscarriage.

**Table 3:** Outcomes of frozen-thawed day 3 cleavage stage embryo transfer cycles.

Outcomes	Group P3 (n=73)	Group P4 (n=87)	*P*
Primary outcome			
Clinical pregnancy (%)	23(31.5%)	28(32.2%)	0.927^b^
Secondary outcomes			
Biochemical pregnancy (%)	29(39.7%)	37(42.5%)	0.720^b^
Implantation (%)	33/136(24.3%)	34/166(20.5%)	0.431^b^
Live birth (%)	22/73(30.1%)	25/87(28.7%)	0.846^b^
Early pregnancy loss(%)	6/73(8.2%)	9/87(10.3%)	0.646^b^
Miscarriage (%)	1/23(4.3%)	1/26(3.6%)	0.440^b^

^a^Mann–Whitney U test, ^b^Pearson’s Chi squared test.

**Table 4:** Outcomes of frozen-thawed blastocysts transfer cycles.

Outcomes	Group P5 (n=70)	Group P6 (n=123)	*P*
Primary outcome			
Clinical pregnancy (%)	41(58.6%)	77(62.6%)	0.581^b^
Secondary outcomes			
Biochemical pregnancy (%)	45(68.6%)	81(65.9%)	0.826^b^
Implantation (%)	62/133(46.6%)	107/231(46.3%)	0.956^b^
Live birth (%)	35/70(50.0%)	66/123(53.7%)	0.625^b^
Early pregnancy loss(%)	6/70(8.6%)	10/123(8.1%)	0.915^b^
Miscarriage (%)	5/40(12.5%)	10/77(13.0%)	0.940^b^

^a^Mann–Whitney U test, ^b^Pearson’s Chi squared test.

Baseline demographics and cycle characteristics were compared between patients who achieved clinical pregnancy after frozen-thawed Day 3 cleavage-stage embryo transfer (**Table 5**). Patients who achieved pregnancy after the transfer had a mean duration of 3.5 ± 0.5 days (range: 3–4 days) of progesterone administration before FET. After controlling for age, body mass index, endometrial thickness at transfer, whether the embryo had a morphology grade of ≥730, and the number of days of progesterone administration, the odds of achieving a clinical pregnancy were not modified (odds ratio [OR] 1.048; 95% confidence interval [CI], 0.518–2.119; *P* = 0.897), **Table 5**).

**Table 5:** A comparison of baseline demographic and characteristics according to whether patients achieved a clinical pregnancy after frozen day 3 cleavage embryo transfer.

Demographics and cycle characteristics	Clinical pregnancy (n=51)	Non clinical pregnancy (n=109)	Adjusted OR (95%CI)	*P*
Female age (year)	31.6 ± 5.1	33.6 ± 5.7	0.935 (0.874-1.000)	0.049
No. of good-quality embryos transferred	1.6 ± 0.6	1.3 ± 0.7	1.771 (1.016-3.085)	0.044
Endometrial thickness (mm)	10.2 ± 1.7	9.5 ± 2.2	1.206 (1.019-1.427)	0.029
BMI (kg/m^2^)	23.3 ± 2.7	23.0 ± 3.5	1.05 (0.946-1.165)	0.358
Duration of progesterone supplementation	3.5 ± 0.5	3.5 ± 0.5	1.048 (0.518-2.119)	0.897

CI, confidence interval; OR, odds ratio.

The baseline demographics and cycle characteristics were compared between patients who achieved clinical pregnancy after frozen blastocyst transfer (**Table 6**). Patients who achieved pregnancy after the transfer had a mean duration of 5.6 ± 0.5 days (range: 5–6 days) of progesterone administration before FET. After controlling for age, body mass index, endometrial thickness at transfer, embryonic day of development at freezing, whether the embryo had a morphological grade of 3BB or better, and the number of days of progesterone administration, the odds of achieving clinical pregnancy were not modified (OR 1.339; 95% CI, 0.717–2.497; *P* = 0.36), **Table 6**).

**Table 6:** A comparison of baseline demographic and characteristics according to whether patients achieved a clinical pregnancy after frozen blastocyst transfer.

Demographics and cycle characteristics	Clinical pregnancy (n=118)	Non clinical pregnancy (n=75)	Adjusted OR (95%CI)	*P*
Female age (year)	30.6 ± 4.5	31.7 ± 4.2	0.949 (0.885-1.018)	0.142
No. of good-quality embryos transferred	1.4 ± 0.8	1.3 ± 0.8	1.142 (0.769-1.696)	0.509
Endometrial thickness (mm)	10.6 ± 2.5	9.6 ± 2.1	1.21 (1.053-1.390)	0.007
BMI (kg/m^2^)	22.8 ± 3.1	22.6 ± 3.3	1.019 (0.925-1.123)	0.701
Duration of progesterone supplementation	5.6 ± 0.5	5.6 ± 0.5	1.339 (0.717-2.497)	0.36

CI, confidence interval; OR, odds ratio.

## 4 Discussion

HRT for FET cycles includes the sequential administration of exogenous estrogen and progesterone to imitate the physiological hormonal exposure of the endometrium in a normal menstrual cycle and to accurately time the transfer of the thawed embryo to the receptive endometrium (19, 34). In HRT cycles, progesterone is initiated to promote the final phase of endometrial preparation prior to embryo transfer (28, 31). Empirically, progesterone supplementation is usually initiated 3 days before the embryo transfer with excellent pregnancy rates in artificially prepared cycles (35). Most reproductive medicine centers believe that a stable clinical pregnancy rate can be obtained by administering the protocol of transferring the Day 3 embryos with progesterone administration for 3 days and transferring blastocysts with progesterone administration for 5 days in HRT cycles (25, 26, 35). Some reproductive medicine centers still consider that a similar or even higher clinical pregnancy rates can be achieved by prolonging the program by one day (29, 30). These scholars maintained that prolonging the time of endometrial transformation using progesterone can diminish uterine contractions, improve endometrial receptivity, and foreshorten the period between development and implantation after embryo transplantation in the FET cycle (29).

However, the hypothesis that prolonging the duration of progesterone administration could increase the pregnancy rates has not yet been confirmed (29, 30, 36, 37). Better implantation outcomes can be obtained by prolonging the exposure of progesterone to the endometrium during HRT cycles, which prolongs the endometrial receptivity period by increasing the interaction between the embryo and endometrium (38). However, this assumption was not fully confirmed in the present study. Although the clinical pregnancy rates of FET Day 3 embryos on the fifth day of progesterone administration were higher than those on the fourth day of progesterone administration, there was no statistically significant difference between the two groups. This research showed that transferring vitrified-warmed Day 3 cleavage stage embryos on the fifth day of progesterone administration may not increase pregnancy rates compared to transferring on the fourth day of progesterone administration. In addition, this study demonstrated that transferring vitrified-thawed blastocysts on the seventh day of progesterone supplementation may not improve the pregnancy rates compared to transferring blastocysts on the sixth day of progesterone supplementation. To our knowledge, this is the first prospective trial comparing the effects of different duration of progesterone administration before FET. Although various studies have indicated a trend toward better pregnancy outcomes with shorter progesterone supplementation, no definite conclusion has been reached (39–43). Furthermore, it has been proposed that WOI switches approximately 48 h after the initiation of progesterone and is sustained for at least 4 days (44). This study showed that similar pregnancy outcomes were achieved with or without extending progesterone by one day, which may indicate that WOI may be longer than estimated (40, 42). Previous studies have shown that delayed endometrial development during the luteal phase occurs in approximately 25% of the general population. Thus, delaying the day of embryo transfer is feasible (36, 37). Perhaps, the extended day is also theoretically covered by the implantation window (29). This viewpoint seems to have been recognized in the present study.

The hypothesis that prolonging the duration of progesterone administration could diminish the early pregnancy loss rate has not yet been confirmed (42). This hypothesis was not verified in the present study. A higher risk of early pregnancy loss may occur when the duration of progesterone administration before transplantation is shorter than embryonic age (42). Thus, extended progesterone administration before transfer is recommended (45). Implantation after the normal endometrial receptivity period is closely associated with early pregnancy loss (46). A prolonged window of endometrial receptivity may account for the delayed implantation of severely damaged embryos and the subsequent early pregnancy loss (47). A higher incidence of early pregnancy loss was not detected in between group P3, which was administered progesterone for 3 days, and P4, which was administered progesterone for 4 days before transferring vitrified-warmed embryos at the third day cleavage stage, This was not observed in either group P5, which was administered progesterone for 5 days, or P6, which was administered progesterone for 6 days before transferring vitrified-thawed blastocysts, which may be because the duration of progesterone administration was no shorter than the age of the embryo (42). According to these data, there may also be no difference in the rate of early pregnancy loss between the two groups with or without prolonged progesterone administration, which may be due to adaptation to the normal period of endometrial receptivity (46). In contrast, Day 3 embryos of group P4 were transferred on the fifth day of progesterone supplementation, and blastocysts of group P6 were transferred on the seventh day of progesterone supplementation, which might have been too long, although no evidence exists that longer progesterone supplementation had a negative impact on success rates (43).

Based on these results, it may be concluded that enhanced flexibility would be possible when programming vitrified-warmed embryos transfer after progesterone administration, because no differences were observed between the duration of progesterone administration ≥1 day of embryonic age as far as clinical pregnancy, implantation, live birth, and early pregnancy loss rates are concerned (29, 30, 36, 37). This study demonstrated that there may be no correlation between the developmental stage of the blastocyst and clinical pregnancy in the FET cycle in day 5 embryos or day 6 blastocysts when an equal duration of progesterone supplementation was employed, considering that the duration of progesterone supplementation for day 5 and day 6 blastocysts was equal before FET (48, 49). In the light of this result, it could be concluded that clinical pregnancy may not be related to the developmental stage of blastocysts, but rather to the quality of the embryo, as morphologically high-quality day 5 and 6 blastocysts resulted in similar pregnancy outcomes, possibly due to the lack of difference between high-quality embryos on days 5 and 6 regarding aneuploidy (48, 49). However, this assumption may be challenged because other data from some scholars indicated that a blastocyst on day 6 has diverse developmental potential and a specific synchronization between the embryo and endometrium with a different WOI compared with a blastocyst on day 5 (50). On the basis of these results, programming the transfer of a vitrified-warmed blastocyst on day 6 requires further investigation, since these “delayed” blastocysts may encounter different and possibly narrower WOI compared with day 5 embryos. Consequently, the optimal duration of progesterone administration remains controversial.

In addition, this study discovered that blastocysts might have higher pregnancy outcomes in FET cycles compared with that of cleavage-stage embryos (51), which might be related to differences in intimal thickness between them. Additionally, differences in age and the number of good-quality embryos transferred may be related to these pregnancy outcomes as well. An explanation for these findings may be that blastocysts undergo a re-selection procedure that spans the 8-cell-stage developmental block and eliminates embryos with poor developmental potential compared to cleavage embryos (52–54). Moreover, this study found that a stable clinical pregnancy may have no correlation with the number of good-quality embryos, indicating the feasibility of single blastocyst transfer, effectively reducing the incidence of ovarian hyper stimulation syndrome, multiple birth rates, and fetal and maternal risks while ensuring relatively higher pregnancy and live birth rates (55–57). In addition, the current trend in assisted reproduction may become a more feasible approach in extending the duration of *in vitro* embryo culture to obtain more developed embryos, determining the implantation window for these mature embryos, and further improving the success rate of assisted reproduction (29). Single embryo transfer (SET) may be safer than double embryo transfer and as effective as single blastocyst transfer if patients are thoroughly informed of the reasons for the proposition (55, 56). The question may not be whether to apply SET, but how to apply it in terms of patient selection, patient-centered counselling, and coverage of treatment (3).

The data also displayed that the pregnancy outcome of embryos at the cleavage stage might be related to the woman’s age, while the blastocyst pregnancy outcome might be uncorrelated with it (53). It is possible that the aneuploidy rate of oocytes increases with age and then undergoes an embryo developmental block or poor developmental potential (58). However, blastocysts could have better quality and higher euploidy because of a re-selection procedure that spans developmental blocks and eliminates embryos with poor developmental potential compared to that of cleavage embryos (52–54). These results indicate that the difference in pregnancy outcomes between the transfer of blastocysts and day 3 cleavage embryos may largely depend on the quality of the embryos transferred (59).

This study had similar or different results from those of other studies, possibly due to epidemiological variables, limitations of its design, a high level of heterogeneity in the study populations, and insufficient sample size. Although this study adjusted for multiple potential confounding factors, caution should be taken when considering the results as a basis for definitive policy due to the prospective design of a single-center study with a limited sample size, as well as possible differences in research protocol and clinical performance. Consequently, confirmation of these findings in a multi-center study with a large sample size and a more rigorous research design is warranted.

## 5 Conclusion

In summary, although the results of this study need to be confirmed in multicenter randomized trials, they suggest that obtaining as many high-quality blastocysts as possible and programming single blastocyst transplantation deserved consideration. Although the optimal duration of progesterone supplementation remains to be clarified, it is recommended to transfer embryos on days 3 and 5 with a degree of flexibility. However, there may be different and possibly narrower WOI for the blastocyst on the sixth day of vitrification. Further research is required to optimize FET protocols and distinguish other contributing factors that may affect pregnancy outcomes after transferring frozen-thawed embryos.

## Data availability statement

The original contributions presented in the study are included in the article/supplementary material, further inquiries can be directed to the corresponding author.

## Ethics statement

The studies involving human participants were reviewed and approved by the Affiliated Hospital of Southwest Medical University Ethics Committee. The patients/participants provided their written informed consent to participate in this study.

## Author contributions

LL and HZ: conceptualization and writing-original draft prepatation. JH and XS: data curation DL and GH: revising the manuscript critically for important intellectual content. All authors contributed to the article and approved the submitted version.
